# Light, sleep‐wake rhythm, and behavioural and psychological symptoms of dementia in care home patients: Revisiting the sundowning syndrome

**DOI:** 10.1002/gps.5712

**Published:** 2022-04-26

**Authors:** Ta‐Wei Guu, Dag Aarsland, Dominic ffytche

**Affiliations:** ^1^ Departments of Internal Medicine, Division of Psychiatry China Medical University Beigang Hospital Yunlin Taiwan; ^2^ Sleep Medicine Center and Mind‐Body Interface Laboratory (MBI‐Lab) China Medical University Hospital Taichung Taiwan; ^3^ Department of Old Age Psychiatry, Institute of Psychiatry, Psychology and Neuroscience King's College London London UK; ^4^ National Institute for Health Research (NIHR) Maudsley Biomedical Research Centre (BRC) at South London and Maudsley NHS Foundation Trust London UK; ^5^ Centre for Age‐Related Medicine Stavanger University Hospital Stavanger Norway

**Keywords:** Alzheimer's disease, Charles Bonnet syndrome, circadian rhythm, dementia, hallucinations, lighting

## Abstract

**Objectives:**

It is believed that inadequate environmental light, especially in facilities such as care homes, contribute to the diurnal changes of behavioural and psychological symptoms of dementia (BPSD) historically referred to as “sundowning syndrome”. Conceptual models of sundowning phenomena have shifted emphasis from the role of light in vision (image forming) to its role in circadian rhythm modulation. However, the grounds for this change are unclear and the evidence on which it is based has not been examined comprehensively.

**Methods:**

We have searched literature on sundowning syndrome and its association with light and studies evaluating BPSD, behavioural rhythm and environmental light in care homes in four databases (PubMed, Web of Science, Embase and Cochrane Library) from inception to 31 January 2021.

**Results:**

Of the nine studies investigating light, behavioural rhythm and BPSD in care homes identified, we found evidence that insufficient natural light exposure was associated with worsening of BPSD and disrupted activity rhythm but it was not clear whether this related to image forming or disrupted circadian rhythm. There was a paucity of evidence in relation to the role of low levels of light for image forming in the context of a specific BPSD symptom: visual hallucinations. We also found literature on the possible role of light outside the visible spectrum influencing cognition. Based on the evidence, we proposed a new model integrating different components of light in BPSD and sundowning syndrome that combines its image forming and circadian roles.

**Conclusions:**

Inadequate light may be a risk factor for BPSD and sundowning syndrome for dementia patients through a range of different mechanisms. It is recommended that multiple neuro‐endocrinological and socio‐environmental factors relevant to light such as adjusting the environmental setting, increasing light exposure, and scheduling activities should be considered when treating dementia patients with BPSD.

## Key points

- This article explores the evolution of scientific hypothesis about “sundowning syndrome”, or the diurnal‐nocturnal variation of behavioural and psychological symptoms in dementia (BPSD) and tries to elucidate the contribution of environmental light's image forming (IF) and non‐image forming (NIF) functions to the syndrome.
- Studies focussing on inadequate environmental light exposure in care home dementia patients and their behavioural rhythm and BPSD are reviewed. Correcting insufficiency in ambient light might be helpful to alleviate BPSD, which is likely associated with both the IF and NIF functions of light.
- There was a paucity of evidence on the role of IF light in relation to the specific BPSD symptom of visual hallucinations.
- Based on currently available evidence, we propose a novel model for BPSD and sundowning syndrome integrating light's IF and NIF effects and discuss the emerging evidence related to the invisible light spectrum.

## 1 INTRODUCTION

As the population ages worldwide, Alzheimer's disease and related dementias become a significant global burden.^1^ In addition to cognitive decline, the neuropsychiatric symptoms of dementia, also referred to as behavioural and psychological symptoms of dementia (BPSD), add significantly to the morbidity of dementia for both patients themselves and their caregivers.^2^ The prevalence of BPSD has been reported to be higher in long term care facilities, such as care homes or nursing homes than in the community, which may partially be explained by BPSD being more common in advanced dementia stages and sampling bias, that is dementia patients with BPSD are more likely to be institutionalised.^3^, ^4^ However, the possible impact of insufficient illumination level and inadequate light exposure among care home patients as factors in this higher prevalence needs to also be considered.^5^, ^6^, ^7^

In the 1940s, symptoms which would today be classified as BPSD were noted to worsen in the late afternoon or early evening.^8^ It was believed then that “the inability to maintain spatial image under low light level” and “without the assistance of a repeated visualisation” were the major causes of this phenomenon. This association was later referred to as ‘sundowning’ as it was thought the changes in behaviour were caused by the reduced levels of light at sunset.^9^ Although the concept of a sundowning syndrome remains in current use, it is poorly defined and the underlying mechanism poorly understood. One explanation for the behavioural change relates to that proposed for visual hallucinations in the context of eye disease (Charles Bonnet syndrome)^10^, ^11^, ^12^, ^13^ and Parkinson's disease^14^ where reduced ambient light is thought to increase the risk of visual hallucination through decreased input to the visual system. Another more recent account highlights dysregulation of circadian biology and changes in circadian rhythm related to reduced light as the cause.^15^ In support of this circadian account, recent clinical and preclinical studies find evidence that mood, sleep and aggressive behaviours might be regulated by the circadian system that is itself controlled by light.^16^ Inappropriate ambient light and subsequent dysregulated circadian rhythms might thus become a plausible cause for sundowning.

While literature on bright light therapy (or bright light treatment, defined as “enhanced indoor electrical light scheme”^17^) for sleep disorders or BPSD in dementia has recently been reviewed, there has been no previous publication comprehensively examining the existing literature regarding impact from environmental light to BPSD focussing on vulnerable care home populations or the possible contributions of light used for vision (image forming, IF) and light used to control circadian functions (non‐image forming, NIF).

## 2 METHOD

**FIGURE 1:**
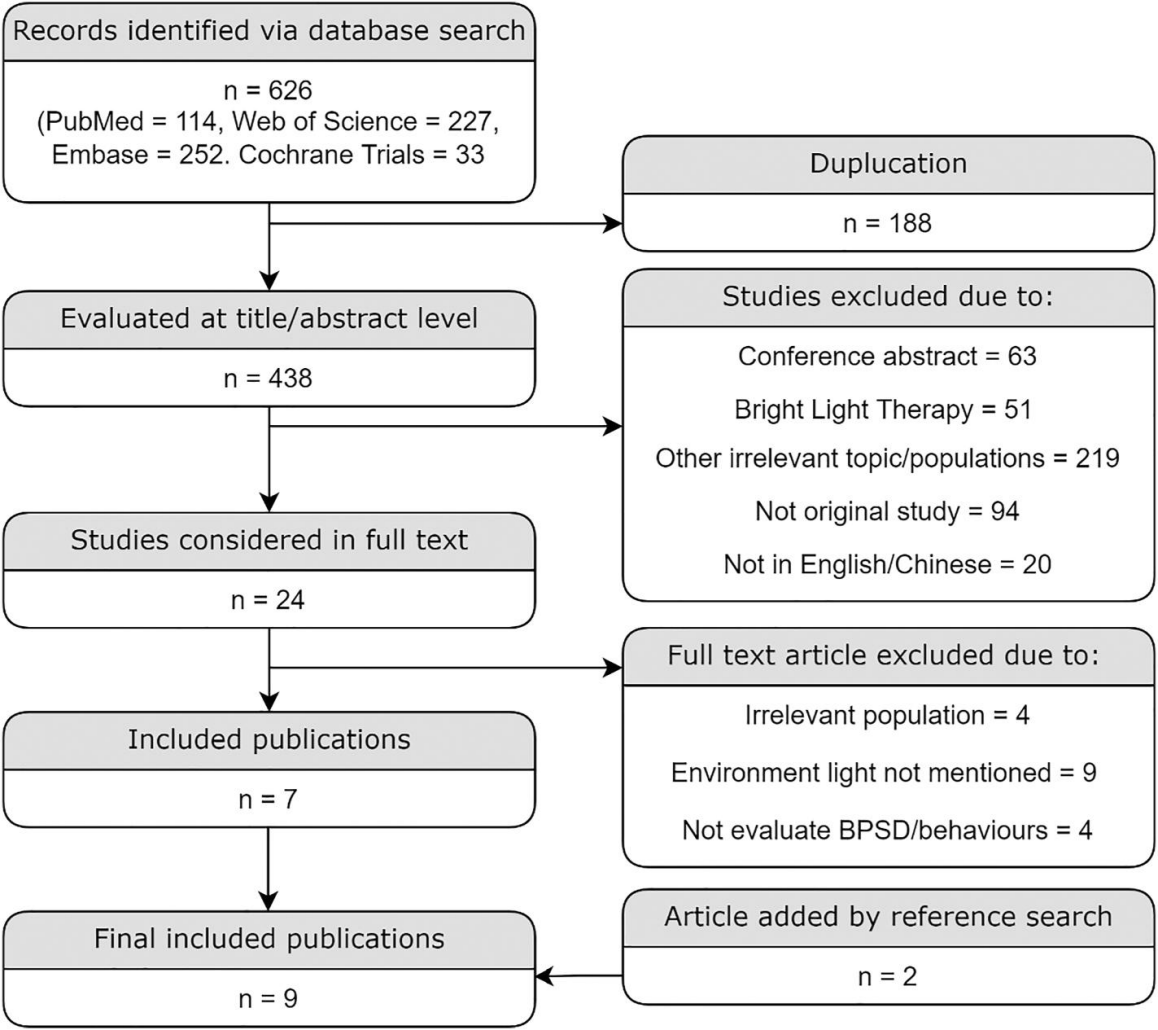
Flow diagram of the search, selection and review process of studies focussing on how ambient light influences behavioural and psychological symptoms of dementia in care home dementia patients

We have searched literature to January 2021 in PubMed, Web of Science, Embase and Cochrane Library using the keywords “care home*” OR “nursing home*” OR “residential home*” AND “behavioural symptom*” OR “circadian rhythm*” OR “sundown*”. No further limits or filters were set. The titles of search results were then screened for original studies specifically exploring the BPSD or rest‐activity rhythm in Alzheimer's disease and related dementias in care home settings. Relevant full text articles were retrieved to evaluate for inclusion. Articles were selected according to following criteria: (1) Cognitive functions or dementia of elderly care home population were assessed by validated criteria, (2) BPSD or behavioural rhythm data were collected using standardised measures, (3) the ambient light conditions were well described and (4) articles were published in English or Chinese language, were peer‐reviewed and with full text available. The reference list of selected publications was reviewed to identify any missing studies. We found 626 publications and initially selected 7 original research publications focussing on the influence of ambient light on BPSD in care home settings and reviews of mechanisms of sundowning. An additional 2 papers^21^, ^23^ were added after looking into the references of the 24 original studies read in full text and review articles cited in the references. Studies primarily focus on bright light therapy are only included here where relevant to the consideration of ambient light as an aetiological factor in BPSD. The search strategies and reasons for excluding 617 studies are shown in Figure 1.

## 3 RESULTS AND DISCUSSION

In what follows we examine studies related to ambient light, BPSD and behavioural rhythms in dementia patients living in care homes. We then move on to describe the visual (IF) and circadian (NIF) functions of visible light and how each might contribute to BPSD, as well as the role of invisible light on cognitive function and dementia. Finally, we propose a novel account of light and behaviour interaction in dementia patients that combines old and new theories of sundowning.

### 3.1 Ambient light, BPSD and behavioural rhythm in care home patients

It may not be surprising that seasonal daylight differences impact human behaviours and affect^18^; however, it was not until recent decades that people started to look at the interplays between environmental light exposure and activity rhythm and/or BPSD in care home patients.^5^, ^19^, ^20^, ^21^, ^22^, ^23^, ^24^, ^25^, ^26^, ^27^ Based on our selection criteria, only seven articles published so far examined the sleep‐wake cycle and/or other BPSD under natural light settings in care homes, including five observational studies and two interventional studies. There were two additional studies evaluating the influence of different natural light conditions in comparison to bright light therapy on BPSD and specifically reported the parameters and outcome under natural light. None of the studies adopted a randomised controlled design. The results are summarised in Table 1.

**TABLE 1:** Overview of the studies evaluating ambient light conditions and behavioural and psychological symptoms of dementia or sleep‐wake rhythm in care home settings

Study [ref.], year, country	Participants	Design; setting and ambient light conditions	Relevant measurement	Main findings
Ancoli‐Israel et al.,^20^ 1997, USA	*N* = 77 (*F* = 58), mean MMSE = 12.8 (severe dementia, *n* = 55), mean age 85.7	Observational cohort study in two nursing homes, natural setting, no intervention	Sleep‐wake behavioural rhythm, light exposure in patients of different degree of dementia, by a wrist‐worn actigraphy for 3 days	Patients with more severe dementia slept more, had more blunted activity rhythms and less bright light exposure
Schnelle et al.,^21^ 1999, USA	*N* = 184 (*F* = 150), mean MMSE: 10.9, mean age 83.9	Four‐phase pre‐and‐post in eight nursing homes, natural setting, interventions not involving physical environment modifications (primarily on noise reduction)	Sleep (7 p.m. to 5 a.m.) by wrist‐worn actigraphy. Light changes and noise assessed separately by a bedside cadmium sulfide photocell and an electric microphone, for 4 nights	Light changes reduced 50% with intervention. Wakes from sleep reduction associated with both light change reduction alone and noise + light change reduction
Shochat et al.,^5^ 2000, USA	*N* = 77 (*F* = 58), mean MMSE = 12.8, mean age 85.7	Observational cohort study in two nursing homes, natural setting, no intervention	Sleep‐wake behavioural rhythm, mean/median light levels, minutes over 1000/2000 lux, by a wrist‐worn actigraphy for 3 days	Mean/median: 485/54 lux; time over 1000/2000 lux: 10.5/4 min. Daytime light consolidated night sleep, predicted peak activity
Song et al.,^22^ 2009, Korea	*N* = 10 (*F* = 10), mean MMSE = 13.1, mean age 85.6	Observational cohort study in a nursing home and an assisted living facility, natural setting, no intervention	Rest‐activity rhythm measured by a wrist‐worn actigraphy. Light measured separately by calibrated precision light metres	Mean p.m. light: 2038 ± 288 lux (common areas)/591 ± 498 lux (bedrooms). All with disturbed rhythm, but influenced by settings for example, scheduled activity
Nioi et al.,^23^ 2017, UK	*N* = 16 (*F* = 13), mean MMSE = 22, age 72–99	Repeated measures design comparing same cohort in summer/winter in six care homes, natural setting, no intervention	Sleep‐wake behavioural rhythm by a wrist‐worn actigraphy, light by another lapel mounted actigraphy for 4 days. Visual, cognitive and mental wellbeing compared	More daylight, bright light exposure and activity in summer. Cognitive function correlated positively with sleep latency and visual function
Wahnschaffe et al.,^24^ 2017, Germany	*N* = 20 (*F* = 19), mean MMSE = 22, mean age 83.8, dementia diagnosed and subtyped with ICD‐10	Retrospective analysis the impact from season and weather of a dynamic lighting study in one nursing home	Rest‐activity rhythm measured by a wrist‐worn actigraphy for 3 years and compared with daily regional weather data, especially cloud amount and day length	Nocturnal restlessness was positively correlated with cloud amount and negatively correlated with day length
Konis et al.,^25^ 2018, USA	*N* = 77 (*F* = 56), mean age 85.2, MMSE all above 10	Two‐arm parallel intervention study in eight dementia care communities, comparing the effect of daylight exposure (8:00–10:00 a.m. within 3 m from windows)	No actigraphy measurement. BPSD measured for 12 weeks with NPI‐NH and CSDD. Light measured separately by a mobile spectrometer able to capture spectral composition of light and transformed to melanopic illuminance (mLux)	Intervention group has significant reduction CSDD score. The reduction was most prominent in probable major depressive disorder patient (CSDD > 10), and positively correlated with average mLux
Bautrant et al.,^26^ 2019, France	*N* = 19 (*F* = 17), MMSE all below 15, mean age 86.3	Pre‐and‐post in one nursing home, adjusting setting (change ceiling/wall colour, enhanced daylight, gradual darkening night light; also music, clock, clothes)	No actigraphy and no quantitative light measurement. Number and duration of BPSD recorded with questionnaires. Risk of fall, cognition and depression compared	No difference in risk of fall, cognition and depression. Reduction of agitation, aggression, screaming and wandering observed
Juda et al.,^27^ 2020, Canada	*N* = 14 (*F* = 12), cog‐ batteries of NIH toolbox (5 scored < 2SD), mean age 82.6	(*Only control part included.) within‐subject cross‐over design, control used natural settings (conventional light) in one of the two adjacent buildings	Sleep‐wake behavioural rhythm and light exposure by wrist‐worn actigraphy for 5 weeks (control). Cognition, depression, fatigue, sleep quality, chronotype measured	There was a large variability in light exposure for all subjects, irrespective of experimental group. Participants with higher morning light exposure had less fragmented rest‐activity rhythms and higher rhythm amplitude, which correlated positively with cognitive performance

Abbreviations: CSDD, Cornell Scale for Depression in Dementia; MMSE, Mini Mental State Examination; NIH, National Institute for Health Research; NPI‐NH, Neuropsychiatric inventory‐Nursing Home version.

One of the first studies exploring light and behavioural rhythm in nursing homes with objective measures was published in 1997 where patients with severe dementia spent as little as 1 min per day under light above 1000 lux which is significantly less compared to patients with better cognitive functions, indicating that bright light exposure may decrease with the severity of dementia.^19^, ^20^ The same group of authors further showed that dementia patients in nursing homes not only had a low level of light exposure, but also found that a higher level of daytime light exposure was associated with less fragmented night sleep, and the acrophase (time of peak) of light exposure significantly correlated with the acrophase of activity level suggesting that the maximum activity occurred at the time of maximum light, typically the middle of the day. Longer exposure of bright light (time spent over 1000 lux) also predicted later activity acrophase, regardless of the severity of dementia.^5^ Schnelle et al. introduced a set of interventions primarily aimed at reducing the environmental noise without physically modifying the environment, such as education sessions to staff, verbal feedback for noises, closing room doors, turning off unwatched television, and moving the curtains more slowly, while also recording the bedside light change times (defined as a change of 1 foot candle or more between successive 2‐min intervals as recorded by the bedside photocell), to see how patients' sleep was influenced.^21^ The temporal resolution of actigraphy measurement used for sleep was relatively low (4 Hz) with less accurate monitoring of movement compared with the resolution commonly used today (usually 20–50 Hz). However, it was sufficient to find the times patients awakened from sleep reduced with the intervention to minimise light changes at night. Song et al published a study in Korea in 2009 with 10 female subjects recruited from two homes,^22^ and found these patients all presented with fragmented night sleep and frequent daytime napping despite adequate light conditions even in the afternoon [2038 ± 288 lux (common areas)/591 ± 498 lux (bedrooms)]. In this study, the author also found the service setting, such as scheduled activities program and meal time, may be able to stabilise rest‐activity rhythm.

Two studies evaluated the impact of natural light on BPSD.^23^, ^24^ A Scottish study evaluated the impact of seasonality.^23^ It found a significant seasonal difference in average morning illuminance level and bright light (over 1000 lux) exposure among 16 care home patients (466 lux/46 min in summer and 65 lux/3 min in winter), but this was not associated with a significant difference in sleep parameters. A German study,^24^ on the other hand, retrospectively analysed the data of a bright light therapy study and compared it with local weather data. The study nursing home was equipped with large windows allowing high daylight fluxes to the interior living environment, and the authors found that both the cloud amount and the day length were significantly correlated with night time restlessness, that is more clouds and shorter length of daylight were both associated with more restlessness.

With proper interior design to introduce adequate daylight, Konis et al. found that even without bright light therapy, exposure to daylight from windows (within 3 m) in the morning (from 8 to 10 o'clock) for 12 weeks could be helpful to reduce subjects' depressive symptoms.^25^ A recent prospective study compared the reduction of BPSD after multiple environmental modifications associated with day‐night orientation.^26^ The modifications included brightening the colours of ceiling and walls, reinforcement of daytime lighting, and progressive lowering the light at night. Although this pre‐and‐post study also applied other environmental factors, such as soothing music, enlarged clocks and changing of staffs' clothes colours and was without direct objective measurement of light exposure and behavioural rhythm, it showed reduction of several dangerous BPSD, including agitation, aggression, wandering and screaming. The final study, adopting a cross‐over, within subject comparison design, used circadian lighting in the intervention phase, which is considered a type of bright light therapy. This study specifically compared individual subjects behavioural rhythms during the 5 weeks control phase with conventional/natural lighting. Regardless of the lighting conditions, the authors found that light exposure varied greatly between participants, that higher morning light exposure was associated with less disrupted rest–activity rhythms, higher intra‐daily rest‐activity rhythm variability and long sleep duration predicted poorer cognitive function.^27^

### 3.2 Light and vision (image forming)

Image forming is the primary and most well‐known function of light. Even in the non‐demented elderly, the light used for image forming is usually reduced due to degenerative changes affecting light transmission in the eye and retinal ganglion cell loss.^28^ Many countries have developed specific regulations to set minimum requirements for environmental light in care homes to ensure the quality of life and safety of the senior citizen living there, with many setting the indoor lighting level to be 150 lux or above in most living areas.^29^, ^30^ Good, diffused and glare‐free lighting in care homes reduces eye strain and provides a safe and comfortable working environment for staff, while inadequate illumination not only hinders proper care, but is itself a risk factor for fall and injury of both the staff and care home residents.^31^

When eye disease becomes severe enough, some elderly patients may develop vivid visual hallucinations, referred to as Charles Bonnet syndrome (CBS), irrespective of the diagnosis or severity of dementia.^32^ This syndrome is associated with degenerative changes of the retina, intraocular light transmission, or pathology of the visual pathways or visual cortex and can interact with the pathophysiology of dementia and BPSD.^33^ Although increasing light levels are widely suggested as treatment for hallucinations across a range of contexts, few studies have explored whether reduced ambient light contributes to the visual hallucinations of CBS and the findings are inconclusive (Table 2).^10^, ^11^, ^12^, ^13^ None of the studies have specifically set out to investigate the effects of light but report the typical ambient lighting conditions that are associated with hallucinations. Each study uses its own descriptive scale for the light conditions making it difficult to compare across studies. Among these studies, one found visual hallucinations were most frequent in dim or low light,^10^ while another study found they were more likely to occur in bright light.^12^ The remaining studies found light had no definite effect for the majority of patients.^11^, ^13^ Patients either reported their visual hallucination were not light dependent, or the proportions of patients reporting visual hallucinations in bright light, dim or no light conditions were very similar. Although the evidence for light enhancement is inconclusive, enhancing environmental lighting combined with a review of cognitive, physical and ophthalmological health are recommended as first line treatment for visual hallucinations in elderly patients.^33^, ^34^

**TABLE 2:** Ambient light level and visual hallucination (VH) in Charles Bonnet syndrome (regardless of the primary ophthalmologic illness)

Study [ref.], year, country	Participants	Study design and measurements	Cognitive and other psychiatric remarks	Main findings on light conditions
Teunisse et al.,^10^ 1994, The Netherlands	*N* = 14 (*F* = 13), age 73–95 years old (mean = 81.8)	Cross section case series. Self‐reported questionnaires followed by MMSE and psychiatry interview	MMSE scores: 10–28 or 25–30 after correction for visual impairment. One with concurrent organic amnestic syndrome and one with dementia. Two with major depressive disorder and two with dysthymic disorder	Facilitated circumstances^a^: Profound darkness: 2 (14%) Poor lighting: 7 (50%) Bright daylight: 1 (7%)
Khan et al.,^11^ 2008, UK	*N* = 97 (*F* = 59), mean age 80.3 years old (SD = 6.0)	Cross section cases (*n* = 97) with non‐VH controls (*n* = 263). Patients were assessed by an ophthalmologist and a visual symptoms questionnaire	Not formally assessed. Education level and percentage of cases and controls with depression and other psychiatric conditions were similar	Precipitating circumstances: Night or low light: 31 (32.0%) Mornings or on waking: 4 (4.1%) None (at any time): 52 (53.6%) Other: 10 (10.3%)
Vukicevic et al.,^12^ 2008, Australia	*N* = 33 (*F* = 25), age 65–92 years old (mean = 77.7)	Cross section cases (*n* = 33) with non‐VH controls (*n* = 30). Referred by ophthalmology clinics for general health, demographic and VH/CBS experience interview	Not formally assessed. 16/33 reported moderate or severe stress as a result of VH. Education level was similar between cases and controls (primary: Secondary education = 79:21 and 80:20)	Light intensity during VH: Darkness: 1 (3%) Dim light: 6 (18.2) Bright light: 20 (60.6%) Unsure: 6 (18.2%)
O’Hare el al.,^13^ 2015, Australia	*N* = 27 (out of 72 advanced retinitis pigmentosa patients)	Part of a larger natural history study of retinitis pigmentosa. Full ophthalmological examination and a CBS questionnaire	No formally assessed. No education or psychiatric conditions/history reported in the study published	Light intensity during VH: Darkness: 3 (11.0%) Dim light: 3 (11.0%) Bright light 6 (22.2%) Both light and dark: 3 (11.0%) Not light dependent 12 (44.7%)

Abbreviations: CBS, Charles Bonnet syndrome; VH, Visual hallucinations.

^a^ Percentage not rounded to 100 as some facilitated circumstances were not relevant to light conditions.

### 3.3 Light and circadian or sleep‐activity rhythms (non‐image forming ‐ NIF)

Light may not only be necessary for vision and image forming, but also has NIF effects on circadian rhythms. The intrinsically photosensitive retinal ganglion cells are believed to regulate the NIF light effects, including melatonin suppression and daily light entrainment (synchronisation) that enhance daytime alertness. These effects derive from light with wavelength range from 446 to 484 nm at the blue/violet end of the spectrum of at least 2500 lux, to stimulate the built‐in biological clock in the suprachiasmatic nucleus and subsequently sending alerting signals to the brain and body for neuroendocrine and neurobehavioural responses.^28^, ^35^, ^36^, ^37^ During night and darkness, the clock receives pineal gland‐produced melatonin leading the brain and body to rest and forming the circadian rhythm. In the elderly, the diurnal light signal input is reduced resulting in circadian disruption due to several factors including ageing and eye disease, degeneration of intrinsically photosensitive retinal ganglion cells and dysfunction of the suprachiasmatic nucleus that receives the light information and controls the biological clock.^28^, ^37^

Because degenerative changes of the human circadian regulating system seem to positively respond to the aforementioned specific wavelength of light, several studies have used bright light therapy of different sources, intensity, amount and duration, either as monotherapy or in combination with other treatment modalities, to treat the BPSD associated with sleep‐activity rhythmicity, agitation, and mood components and have shown inconsistent but in general promising results.^17^, ^38^, ^39^, ^40^ These studies contribute to our understanding of how the NIF effect of light may influence the human circadian rhythm and some BPSD components. For example, a recent meta‐analysis found that study design, such as the control conditions of a study and the day length of the season in which the study is carried out, could be moderating factors influencing the outcomes.^17^ Interestingly, in the published meta‐analysis, agitation reduction in the bright light therapy condition was negatively associated with the increase of day length in one study so that for longer days there was less improvement in agitation. This implies that control conditions with illumination more than 400 lux may be sufficient to improve agitation, therefore nullifying therapeutic effects when compared to bright light treatment in clinical trials. These findings imply that adequate ambient light may itself ameliorate at least some BPSD and sleep‐wake disorders in the elderly and warrant further studies to explore how these factors interact.

### 3.4 The invisible light

Most of the BPSD studies focus on visible light, especially bright light over 1000 lux and in the blue to green light spectrum, as a treatment or an environmental condition influencing IF and NIF functioning to enhance daytime alertness and modify sleep quality.^41^ However, a small number of studies discuss the impact of light outside the visible spectrum. Although invisible light may have no role in BPSD associated with vision or NIF as it is not captured by the visual system, other ways in which invisible light may influence behaviour have been proposed.

Vitamin D deficiency is associated with an increased risk of Alzheimer disease,^42^ and the major active form of vitamin D, vitamin D3, is produced by the skin after absorbing ultraviolet‐B. Some studies have shown treatment effects of either oral vitamin D3 or exposure of ultraviolet‐B for dementia and BPSD.^43^ On the other end of the invisible light spectrum, there are also preliminary studies using near‐infrared photobiomodulation in dementia.^44^ It was initially used in topical treatment for wound healing and skin conditions and recent studies have shown promising results in dementia, with the most popular wavelengths ranging from 800 to 900 nm, either applied transcranially or intra‐nasally. A possible mechanism underlying the effects is that it activates Cytochrome C oxidase, the terminal enzyme in the electron transport chain of mitochondria, to restore cellular energy production. Unlike ultraviolet, longer wavelength lights have better tissue penetration ability, and depending on the wavelength, up to 2% of the energy can be absorbed transcranially to enhance mitochondria function of the cortex. These studies suggest a potential use of non‐visible light in treating dementia and BPSD that might be considered for future studies in care home populations.

### 3.5 A model of light and behaviour interaction: revisiting sundowning phenomenon

**FIGURE 2:**
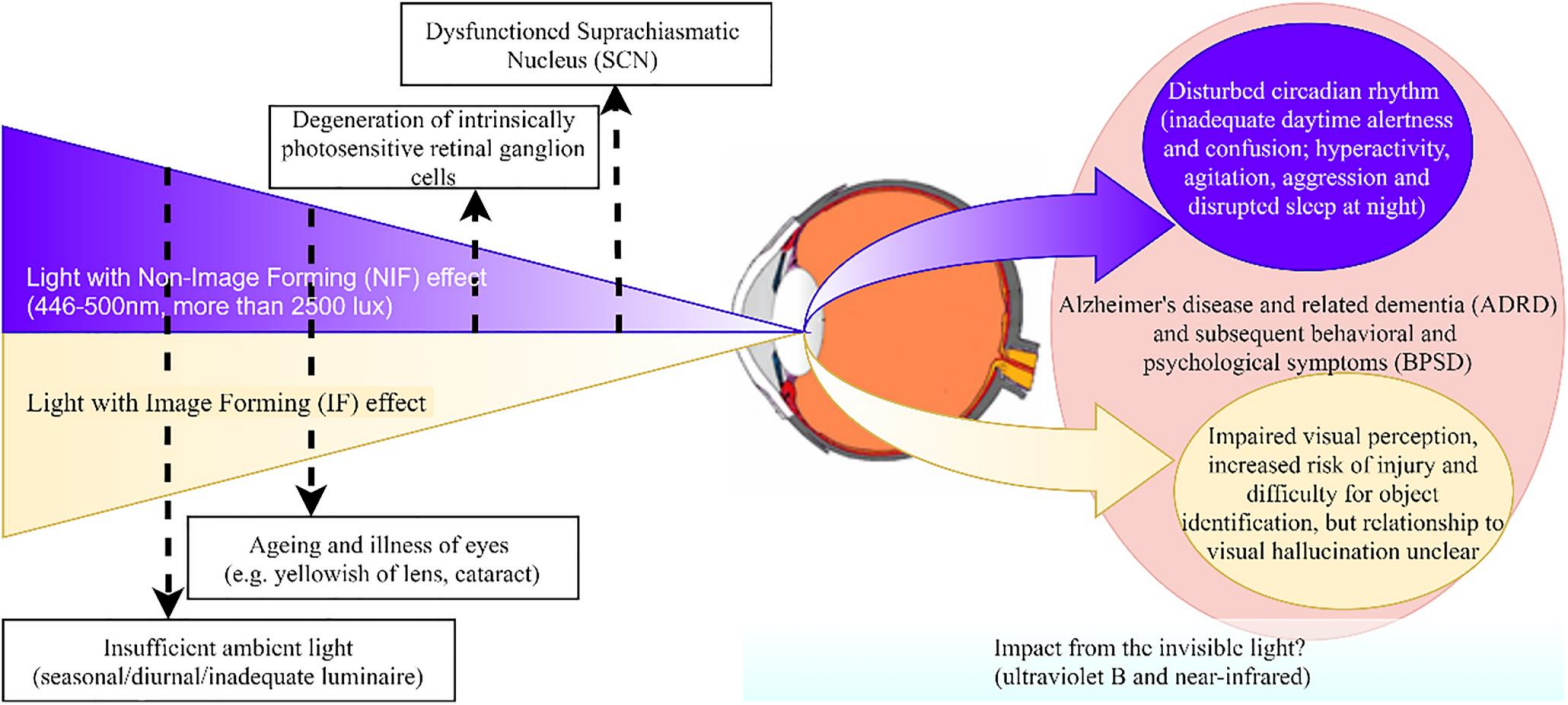
Proposed model of sundowning and the interaction between image forming and non‐image forming light, dementia and behavioural and psychological symptoms of dementia. Light entering the eye is illustrated as a triangle with the non‐image forming component (shaded blue/violet) and the image forming component (shaded beige). The tapering of the triangle indicates attenuation of light by different factors as labelled. The behavioural and perceptual consequences are shaded corresponding to their likely NIF and IF mechanisms

Based on the evidence discussed above, we postulate that both IF and NIF effects of light should be considered in models of BPSD. While inadequate IF light in the environment will impair vision and is thus dangerous to both elderly patients and their care givers in general and might increase the risk of visual hallucinations, NIF light input below the threshold to regulate the circadian rhythm and maintain alertness might lead to disrupted behavioural symptoms such as sleep and agitation. The two visible components of light, and the invisible light are distinct from each other, but could intertwine with the deterioration of cognitive symptoms and behavioural and psychological symptoms in dementia, as depicted in Figure 2.

In the figure light serves both IF and NIF functions and both are affected by various environmental, biological and ageing factors. The degeneration of specific photoreceptors in the retina and neurons in the suprachiasmatic nucleus affects NIF functions. In terms of sundowning, NIF functions may contribute to BPSD through disrupted circadian rhythms while IF functions may contribute through impairing vision, increasing misperceptions and difficulty identifying objects and, possibly, visual hallucinations. There may also be additional effects from the non‐visible spectrum.

Although sundowning can sometimes happen in non‐demented elderly populations, recently published systematic reviews and scoping reviews found it more closely associated with either dementia or decline in cognitive reserve and subsequent compensatory functions.^45^, ^46^ The possible reason behind this association, in addition to the impairing sense of orientation due to poor memory, may be that the suprachiasmatic nucleus receives cholinergic neuron projections from both the brain stem nuclei and cholinergic forebrain that are likely to degenerate in dementia. The alteration of acetylcholine modulated suprachiasmatic nucleus function as found in many pre‐clinical studies of dementia may explain why simply supplementing melatonin to reset the circadian clock may not improve sleep and behavioural disorders in dementia patients.^15^, ^16^ In general, published clinical studies on the treatment of sleep or behavioural symptoms in dementia populations have not found melatonin to be efficacious.^47^, ^48^ Although melatonin is well tolerated in the subjects and does not impair either questionnaire‐based or actigraphy‐based sleep outcome measures, it did not improve sleep structure or subjective sleep quality. A metanalysis of pharmacotherapy for sleep in dementia patients did not recommend the use of melatonin.^49^ It may be the efficacy of melatonin therapy depends on its dose, timing (adjusted according to the individual's core body temperature), and duration, as well as the interplay between acetylcholine neurons and the suprachiasmatic nucleus which, in turn, relates to the severity of dementia and underlying advanced brain pathology.

Our model builds on previous accounts of sundowning by adding in the IF light functions and consideration of the possible role of invisible light. A 2012 systematic review found that several studies reported a significant relationship between environmental light intensity and the development of sundowning syndrome, but the overall results were mixed.^46^ A more recent scoping review reported similar finding regarding light and BPSD, and interestingly, the most prevalent symptom cluster was “psychomotor (agitation, aggression, and restlessness)” followed by “cognitive disturbance (confusion, disorientation and wandering)” not the sleep cluster *per se*.^45^ It is therefore hypothesised several pathways link to sundowning‐related behavioural aggression as shown in both pre‐clinical and clinical studies.^16^ It suggested although the suprachiasmatic nucleus would be the major structure receiving light signal input, its downstream sub‐paraventricular zone actually gates the driver of aggressive behaviours, the ventromedial hypothalamus, creating the suprachiasmatic nucleus‐sub‐paraventricular zone‐ventromedial hypothalamus pathway. Furthermore, as ventromedial hypothalamus may also associate with fear and anxiety regulation, it would be possible this pathway also regulates the emotional component of BPSD.

Our model is primarily intended as a brain and light‐based account of sundowning, but this is not to say that other socio‐cultural factors play no role. For example, it has been argued that factors such as fewer staff and lack of routines around evening time in care homes may contribute to sundowning. It is also possible that psychological factors such as a feeling of being lost as it becomes dark, or fatigue of both patients and caregivers later in the day may associate with the symptoms.^15^ Although sundowning syndrome remains poorly defined, it is likely that multiple neuro‐endocrinological as well as socio‐environmental factors relevant to light, including the visual system and suprachiasmatic nucleus degeneration, melatonin reduction, sensory deprivation, and insufficient light exposure, all play a role.

## 4 CONCLUSION

Light plays an important role in Alzheimer's disease and related dementias not only in its image forming function, creating a safe and enjoyable space for elderly patients and care home staff, but also NIF effects contributing to the sleep‐wake cycle and BPSD in the elderly and, in its invisible spectral components, may interact with the ageing process.

Preliminary evidence with relatively small sample sizes and usually short observation periods has shown that insufficient illuminance or reduced bright light exposure in care homes may correlate with more fragmented sleep, less daytime activities, and is associated with worse cognitive function. Improving light conditions may reduce some BPSD. In this review, the fact that only two non‐randomised interventional studies were identified suggests more well‐designed studies are required to directly elucidate the relationship between ambient light, circadian rhythm, sleep and other BPSD in care home settings. Future studies should focus on how patient characteristics such as baseline chronotype, visual and cognitive function play their roles in BPSD, as well as how to optimise individual patients' behavioural rhythms and light exposure with appropriately designed care home light sources and service schedules.

## CONFLICT OF INTEREST

The authors have nothing to declare.

## Data Availability

The data that support the findings of this study are available from the corresponding author upon reasonable request.
